# Safety and efficacy of direct oral anticoagulation in patients with and without radiofrequency ablation of non-valvular atrial fibrillation: a multicenter retrospective cohort study

**DOI:** 10.1186/s12959-023-00483-6

**Published:** 2023-04-05

**Authors:** Shuyi Wu, Chengfu Guan, Wenlin Xu, Feilong Zhang, Nianxu Huang, Xia Chen, Wang Zhang, Wei Hu, Jun Su, Hengfen Dai, Ping Gu, Xiaohong Huang, Xiaoming Du, Ruijuan Li, Qiaowei Zheng, Xiangsheng Lin, Yanxia Zhang, Lang Zou, Yuxin Liu, Min Zhang, Xiumei Liu, Zhu Zhu, Jianjun Sun, Shanshan Hong, Weibin She, Jinhua Zhang

**Affiliations:** 1 Department of Pharmacy, Fujian Maternity and Child Health Hospital College of Clinical Medicine for Obstetrics & Gynecology and Pediatrics, Fujian Medical University, #18 Daoshan Road, Fuzhou, 350001 China; 2 Department of Cardiology, Fujian Medical University Union Hospital, Fujian, 350001 China; 3 Department of Pharmacy, Taikang Tongji(Wuhan) Hospital, Wuhan, 430000 China; 4 Department of Pharmacy, Fuling Hospital of Chongqing University, Chongqing, 408099 China; 5 Department of Pharmacy, The First People’s Hospital of Changde City, Hunan, 415000 China; 6 Department of Pharmacy, Xinyang Central Hospital, Xinyang Hospital Affiliated to Zhengzhou University, Henan, 464000 China; 7 Department of Pharmacy, The First Affiliated Hospital of Bengbu Medical College, Anhui, 233004 China; 8 Department of Pharmacy, Affiliated Fuzhou First Hospital of Fujian Medical University, Fujian, 350009 China; 9 Department of Pharmacy, Suining Central Hospital, Suining, 629000 Sichuan China; 10 Department of Pharmacy, Zhangzhou Affiliated Hospital of Fujian Medical University, Fujian, 363000 China; 11 Department of Pharmacy, Shengjing Hospital of China Medical University, Shenyang, 110004 China; 12 Department of Pharmacy, Shanxi Bethune Hospital, Shanxi Academy of Medical Sciences, Tongji Shanxi Hospital, Third Hospital of Shanxi Medical University, Shanxi, 030032 China; 13 Department of Pharmacy, First Affiliated Hospital of Xi’an Jiaotong University, Xi’an, 710061 Shaanxi China; 14 Department of Pharmacy, Pingtan County General Laboratory Area Hospital, Fujian, 350400 China; 15 Department of Pharmacy, The First Affiliated Hospital of Jiamusi University, Heilongjiang, 154002 China; 16 Department of Pharmacy, Second Affiliated Hospital, Army Medical University, Chongqing, 400037 China; 17 Department of Pharmacy, Huaihe Hospital of Henan University, Henan, 475000 China; 18 Department of Pharmacy, Affiliated Qingdao Third People’s Hospital, Qingdao University, Shandong, 266041 China; 19 Department of Pharmacy, of People’s Hospital He’nan University of Chinese Medicine (People’s Hospital of Zhengzhou), Zhengzhou, 450003 China; 20 Department of Pharmacy, The Second Affiliated Hospital of Soochow University, Jiangsu, 215004 China; 21 Department of Pharmacy, Affiliated Hospital of Inner Mongolia Medical University, Hohhot, 010050 Inner Mongolia China; 22 Department of Pharmacy, Quanzhou First Hospital, Fujian, 362000 China; 23 Department of Medical Administration, Dongguan Kanghua Hospital, Guangdong, 523000 China

**Keywords:** Radiofrequency ablation, Direct oral anticoagulants, Non-valvular atrial fibrillation, Major bleeding, Thrombosis

## Abstract

**Background:**

Based on the few available studies on the prognostic benefit of using direct oral anticoagulants (DOACs) after atrial fibrillation (AF) ablation. Therefore, this study aimed to evaluate the prognostic differences between patients who underwent radiofrequency ablation (RFA) and those without RFA taking DOACs.

**Methods:**

This is a multicenter retrospective cohort study enrolling 6137 patients with non-valvular AF (NVAF) at 15 hospitals in China. Patient information was collected through a mean follow-up of 10 months and medical record queries. Clinical outcomes included major bleeding, total bleeding, thrombosis, all-cause death, and a composite endpoint of bleeding, thrombosis, and all-cause death.

**Results:**

After adjusting for confounders and propensity score matching (PSM), patients with RFA of NVAF had a significantly lower risk of major bleeding [OR 0.278 (95% CI, 0.150-0.515), *P<*0.001], thrombosis [OR 0.535 (95% CI, 0.316-0.908), *P=*0.020] and the composite endpoint [ OR 0.835 (95% CI, 0.710-0.982), *P=*0.029]. In the RFA PSM cohort, dabigatran was associated with reduced all-cause death in patients with RFA of NVAF [OR 0.420 (95% CI, 0.212-0.831), *P=*0.010]. In the no RFA PSM cohort, rivaroxaban was associated with a reduction in major bleeding [OR 0.521 (95% CI, 0.403-0.673), *P<*0.001], total bleeding [OR 0.114 (95% CI, 0.049-0.266), *P<*0.001], and the composite endpoint [OR 0.659 ( 95% CI, 0.535-0.811), *P<*0.001].

**Conclusion:**

Among patients with NVAF treated with DOACs, RFA was a negative correlate of major bleeding, thrombosis, and composite endpoints but was not associated with total bleeding or all-cause mortality.

**Supplementary Information:**

The online version contains supplementary material available at 10.1186/s12959-023-00483-6.

## Introduction

Atrial fibrillation (AF) is a common arrhythmia worldwide and is a major cause of cardiogenic ischemic stroke and systemic thrombosis (SE) [1, 2]. Patients with AF have a higher incidence of stroke and thromboembolic events than patients with sinus rhythm, significantly impacting morbidity and mortality [3]. Prevent thromboembolism, oral anticoagulants (OAC) are one of the preferred treatments for patients at risk of thromboembolism [4].

Catheter ablation (CA) is the most effective treatment to prevent the recurrence of AF [5], and it has made significant advances in safety and efficacy over the past decade [6–10]. However, CA is also at risk of thrombotic and bleeding events in the perioperative [11, 12]. In patients undergoing CA, perioperative complications are approximately 4%-14%, of which 2%-3% may be life-threatening [13]. A global survey on perioperative complications also showed that major complications occurred in 4.5% of patients treated with CA for AF (2.8% for major bleeding and 0.94% for thromboembolic events) [14]. Therefore, continued anticoagulation with OAC is recommended in the perioperative period of AF ablation [15, 16]. And several trials have shown that using uninterrupted perioperative anticoagulant warfarin or DOACs reduces the incidence of thromboembolic events without increasing the risk of major bleeding [17–20].

In addition, the 2020 European Society of Cardiology (ESC) guidelines recommend anticoagulation for at least 2 months after CA, which should be determined based on the patient's stroke risk profile [13]. Reducing the burden of AF with CA may help reduce ischemic stroke risk [21]. However, anticoagulation afterward requires attention to the adverse events associated with DOACs, including serious bleeding. Therefore the results of studies on the clinical benefits of continuous DOAC therapy need to be followed.

Based on the lack of available studies on the prognostic benefit of using DOACs after ablation. Therefore, this study aimed to evaluate the prognostic differences between patients who underwent radiofrequency ablation (RFA) and those without RFA taking DOACs.

## Method

### Study design

From January 2016 to December 2020, we conducted a multicenter retrospective registration at 15 centers in China (Supplementary Table 1). Supplementary Fig. 1 shows the distribution map of multicenter hospitals. The study registration number is ChiCTR2000031909. The inclusion criteria for this study were as follows: (1) age ≥18 years old; (2) diagnosis of NVAF; (3) treatment with DOACs; (4) patients with known ablation status and follow-up data during hospitalization at baseline. Exclusion criteria were: (1) patients with valvular AF; (2) taking warfarin or aspirin after discharge from the hospital. (3) Patients with incomplete basic data such as age and sex. Since apixaban has no indication for NVAF in China and edoxaban will only be available in China in 2019, the DOACs used in multicenter hospitals are rivaroxaban and dabigatran. A total of 6137 patients with NVAF treated with DOACs were eligible for this study after meeting the inclusion criteria. Of these, 2661 patients underwent radiofrequency ablation (RFA) during hospitalization, and 3476 did not undergo RFA.

### Data collection and definition

Demographic information was collected through the hospital information system, and clinical events were obtained through follow-up visits to patients or their relatives. We collected basic statistics such as age, sex, height, weight, smoking and alcohol consumption, information on comorbid diseases such as hypertension, diabetes, coronary heart disease, vascular disease, liver and kidney insufficiency, and biochemical indices such as total bilirubin, glutathione, glutamic aminotransferase, and creatinine. Information on thrombotic and bleeding events and all-cause deaths after patients took DOACs was collected through follow-up. Based on the patient's clinical information, we performed the CHA2-VASC score [22] (congestive heart failure/left ventricular insufficiency, hypertension, age ≥75 years, diabetes mellitus, stroke or transient ischemic attack (TIA), vascular disease, age 65-74 years and sex as female) and the HAS-BLED score [23] (hypertension, abnormal liver function, abnormal renal function, stroke, bleeding, age >65 years, drugs, alcohol).

### Study outcomes

The primary outcomes of this study were major bleeding, total bleeding, thrombosis, all-cause death, and the composite endpoint of total bleeding, thrombosis, and all-cause death. The International Society on Thrombosis and Haemostasis (ISTH) defines hemorrhage as bleeding leading to death, occurring in a critical organ (intracranial, intra-spinal, intra-ocular, retroperitoneal, intra-articular or intra-pericardial, intra-muscular with fascial compartment syndrome) or with a decrease in hemoglobin level of at least 2 g/dl or transfusion of at least 2 units of red blood cells [24]. Total bleeding includes all bleeding events, including major and minor bleeding. Thrombotic events include stroke, venous thromboembolism, and other sites of thrombosis. Venous thromboembolism was defined as any symptomatic or incidental finding of lower or upper extremity proximal deep vein thrombosis, any non-fatal symptomatic or incidental pulmonary embolism, and death associated with pulmonary embolism [25].

### Statistical analysis

Descriptive statistics are expressed as mean ± standard deviation (SD) for continuous variables and as counts or percentages for discrete variables. Continuous variables were tested for normality, t-tests were used to compare the differences in continuous variables between the two groups of patients if they conformed to a normal distribution; if they did not conform to a normal distribution, non-parametric statistical tests were used. The chi-square test was used to compare the distribution of categorical variables. Logistic regression was used to analyze potential confounders affecting major bleeding, total bleeding, thrombosis, all-cause mortality, and composite endpoints, and covariates included: age, sex, body mass index (BMI), smoking, alcohol consumption, hypertension, diabetes mellitus, heart failure, coronary artery disease, renal insufficiency, hepatic insufficiency, vascular disease, Total Bilirubin (TBIL), Aspartate transaminase (AST), Aspartate transaminase (ALT), creatinine, Platelet count (PLT), Hemoglobin (Hb), antiplatelet agents, Proton pump inhibitor (PPI), H2-blockers, statin, amiodarone, Proton pump inhibitor (ACEI), Angiotensin receptor blockers (ARB), β-receptor antagonists, Calcium Calcium Entry Blockers (CCB), diltiazem, and digoxin.

For further analysis, logistic regression of the variables in Supplementary table 2 was performed to obtain propensity score matching (PSM) to create comparable no-RFA and RFA cohorts in a 1:1 ratio. Nearest neighbor matching was performed using a caliper of 0.02 on the propensity score scale [26]. Since the propensity-matched dataset is a resampling from a sample representing the total, it is a sample within a sample. Whereas the hypothesis test corresponds to the aggregate in which the sample is located, the reduced sample size of the data after PSM would have resulted in a larger *p*-value. Therefore, standardized differences rather than statistical tests were used to assess the balance of covariates within the matched cohort. Standardized differences less than or equal to 0.1 indicate an adequate balance between groups [27]. If a covariate is unbalanced, we will check whether including it in the regression will affect the results.

Statistical analyses were performed using SPSS Statistics v. 22 (IBM Corporation, Armonk, NY, USA) and plotted using R software (R (4.1.1, R Core Team (2021)). Two-sided *P* values <0.05 were considered statistically significant.

## Result

### Baseline characteristics

**Fig. 1:**
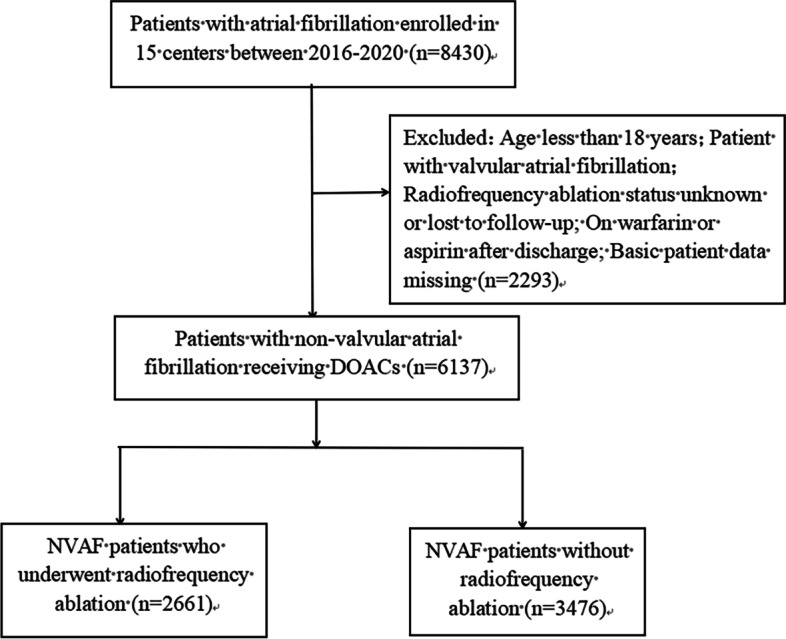
Selection of the study population

A total of 6137 patients with NVAF [3619 (58.9%) male; mean age 63.7 ± 11.6] were included in this study, of whom 2661 patients [1734 (65.2) male; mean age 59.3 ± 10.5] underwent RFA at baseline and 3476 patients without RFA [1885 male (54.2); mean age 67.1 ± 11.3]. The inclusion flow chart is shown in Fig. 1.

Table 1 shows the baseline information table of the patients. Compared with patients who did not receive RFA for NVAF, the group receiving RFA was younger, had more male patients, a slightly higher BMI, a lower burden of comorbid hypertension, diabetes, heart failure, coronary artery disease, hepatic and renal insufficiency, and vascular disease, and lower TIBL, AST, and creatinine values. Patients who underwent RFA had a lower risk of thrombosis and major bleeding than those who did not undergo RFA of NVAF [CHA2DS2-VASc mean 1.9 ± 1.2, HAS-BLED mean 1.3± 0.9]. Regarding combined medications, patients in the RFA group received more PPIs, amiodarone, and ACEIs and fewer antiplatelet agents, statins, ARBs, beta-blockers, diltiazem, and digoxin.

**Table 1:** Patient characteristics at baseline

	No RFA (*n=*3476)	RFA (*n=*2661)	*P* value
Age, years, mean (SD)	67.1(11.3)	59.3(10.5)	<0.001
Sex, male, n (%)	1885(54.2)	1734(65.2)	<0.001
BMI, mean (SD)	24.4(3.8)	24.8(3.3)	<0.001
Smoking, n(%)	2199(63.3)	1730(65.0)	0.003
Alcohol, n(%)	1460(42.0)	1104(41.5)	0.332
Comorbidities, n(%)			
Hypertension	2039(58.7)	1262(47.4)	<0.001
Diabetes mellitus	720(20.7)	361(13.6)	<0.001
Congestive heart failure	199(5.7)	10(0.4)	<0.001
Coronary heart disease	307(8.8)	29(1.1)	<0.001
Renal insufficiency	271(7.8)	25(0.9)	<0.001
Hypohepatia	87(2.5)	14(0.5)	<0.001
Vascular disease	593(17.1)	60(0.23)	<0.001
Laboratories			
TBIL, umol/L, mean (SD)	17.1(9.1)	15.4(6.0)	<0.001
ALT, IU/L, mean (SD)	28.4(41.8)	25.8(19.2)	0.332
AST, IU/L, mean (SD)	45.5(66.2)	35.4(28.8)	<0.001
Creatinine, umol/L, mean (SD)	85.3(30.2)	78.3(17.3)	<0.001
PLT, 10^9^/L, mean (SD)	183.2(65.8)	187.5(50.6)	<0.001
Hb, g/L, mean (SD)	132.0(17.2)	138.3(14.5)	<0.001
Combined medication, n(%)			
Antiplatelet drugs	1009(29.0)	676(25.4)	0.002
PPI	2166(62.3)	1971(74.0)	<0.001
Statins	1902(54.7)	1187(44.6)	<0.001
Amiodarone	557(16.0)	527(19.8)	<0.001
H2-blockers	1098(31.6)	843(31.7)	0.939
ACEI	877(25.2)	575(31.6)	0.001
ARB	950(27.3)	643(24.2)	0.005
β-Blockers	2338(67.3)	1739(65.3)	0.006
CCB	1110(31.9)	829(31.1)	0.515
Diltiazem	556(16.0)	392(14.7)	0.174
Digoxin	419(12.1)	240(9.0)	<0.001
CHA2DS2-VASc, Mean(SD)	2.5(1.5)	1.9(1.2)	<0.001
HAS-BLED, Mean(SD)	1.5(0.9)	1.3(0.9)	<0.001

*RFA* Radiofrequency ablation, *SD* Standard deviation, *BMI* Body mass index, *TBIL* Total bilirubin, *ALT* Alanine transaminase, *AST* Aspartate transaminase, *PLT* Platelet count, *Hb* Hemoglobin, *PPI* Proton pump inhibitor, *ACEI* Angiotensin converting enzyme inhibitor, *ARB* Angiotensin receptor blockers, *CCB* Calcium calcium entry blockers

### Outcomes

During a mean follow-up of 10 months, there were 670 bleeding events (10.9%), including 113 major bleeding events (1.8%), 83 thrombotic events (1.4%), 251 all-cause deaths (4.1%), and a composite endpoint of 979 (16.0%). The specific outcomes for the RFA and no RFA groups are shown in Table 2.

**Table 2:** Clinical Outcomes of RFA vs. no RFA in NVAF patients taking DOACs

	No RFA (*n=*3476)	RFA (*n=*2661)	OR(95%CI)	*P* value	Adjusted OR(95%CI)^b^	*P* value
Total bleeding, n(%)	405(11.7)	265(9.9)	0.839(0.712-0.988)	<0.001	0.901(0.746-1.087)	0.275
Major Bleeding, n(%)	98(2.8)	15(0.6)	0.195(0.113-0.337)	0.035	0.278(0.150-0.515)	<0.001
Thrombosis, n(%)	58(1.7)	25(0.9)	0.559(0.349-0.896)	0.014	0.535(0.316-0.908)	0.020
All-cause death, n(%)	178(5.1)	73(2.7)	0.523(0.396-0.690)	<0.001	0.842(0.611-1.160)	0.293
Composite outcome^a^, n(%)	624(18.0)	355(15.7)	0.704(0.611-0.810)	<0.001	0.835(0.710-0.982)	0.029

*NVAF* Non-valvular atrial fibrillation, *RFA* Radiofrequency ablation, *OR* Odds ratio, *95%CI* 95% Confidence interval

^a^Composite outcome included all-cause death, thrombosis and total bleeding

^b^Adjust for radiofrequency ablation, sex (male), age, body mass index, smoking, alcohol, hypertension, diabetes mellitus, heart failure, coronary artery disease, renal insufficiency, hypohepatia, vascular disease, total bilirubin, ghrelin, glutamic aminotransferase, creatinine, platelet count, hemoglobin, antiplatelet agents, proton pump inhibitors, H2 receptor antagonists, statins, amiodarone, angiotensin-converting enzyme inhibitors, angiotensin receptor blockers, beta-blockers, digoxin, calcium blockers, diltiazem

#### Bleeding events

The incidence of total bleeding was 9.9%, and major bleeding was 0.6% in the RFA group, and the incidence of total bleeding was 11.7%, and major bleeding was 2.8% in the no RFA group. The incidence of total bleeding [OR 0.839 (95% CI, 0.712-0.988), *P<*0.001] and major bleeding [OR 0.195 (95% CI, 0.113-0.337), *P=*0.035] was significantly lower in the RFA group compared with the no RFA group. After adjusting for confounding factors, the risk of total bleeding in patients with RFA was not significantly different from the no RFA group [OR 0.901 (95% CI, 0.746-1.087), *P=*0.275], but the risk of major bleeding remained significantly lower than in the no RFA group [OR 0.278 (95% CI, 0.150-0.515), *P<* 0.001]. The effects of RFA and potential risk factors on total bleeding in patients with NVAF are shown in Supplementary Fig. 2. Patients with combined coronary artery disease and higher creatinine are risk factors for total bleeding in NVAF patients, and combined use of β-blockers and CCB are protective factors. Supplementary Fig. 3 shows the effect of RFA and potential risk factors on major bleeding in patients with NVAF. Greater age, alcohol, combined diabetes mellitus, higher creatinine, and combined use of antiplatelet agents were risk factors for major bleeding in patients with NVAF. Protective factors were RFA, higher BMI, combined vascular disease, higher AST, combined PPI, H2-blockers, statins, ARB, β-blockers, and CCB.

#### Thrombosis events

The incidence of thrombosis was 0.9% in the RFA group and 1.7% in the no RFA. The incidence of thrombosis [OR 0.559 (95% CI, 0.349-0.896), *P=*0.014] was significantly lower in the RFA group compared with the no RFA group. After adjusting for confounding factors, the risk of thrombosis remained significantly lower in patients with RFA of NVAF than in the no RFA group [OR 0.535 (95% CI, 0.316-0.908), *P=*0.020]. The effects of RFA and potential risk factors on thrombosis in patients with NVAF are shown in Supplementary Fig. 4. Alcohol consumption was a risk factor for thrombosis in NVAF patients, and RFA was a protective factor.

#### All-cause death

The incidence of all-cause death [OR 0.523 (95% CI, 0.396-0.690), *P<*0.001] was significantly lower in the RFA group compared with the no RFA group. After adjusting for confounding factors, the risk of all-cause death in patients with RFA of NVAF was not significantly different from the no RFA group [OR 0.842 (95% CI, 0.611-1.160), *P=*0.293]. The effects of RFA and potential risk factors on all-cause death in patients with NVAF are shown in Supplementary Fig. 5. Male, greater age, combined diabetes mellitus, vascular disease, higher TBIL, and combined use of statin were risk factors for all-cause death in patients with NVAF, and higher BMI was a protective factor.

#### Composite outcome

The incidence of the composite endpoint [OR 0.704 (95% CI, 0.611-0.810), *P<*0.001] was significantly lower in the RFA group compared with the no RFA group. After adjusting for confounding factors, the risk of the composite endpoint remained significantly lower in patients with RFA of NVAF than in the no RFA group [OR 0.835 (95% CI, 0.710-0.982), *P=*0.029]. The effects of RFA and potential risk factors on the composite endpoint in patients with NVAF are shown in Supplementary Fig. 6. Comorbid heart failure and vascular disease are risk factors for the composite endpoint in patients with NVAF. RFA, higher BMI, and combined use of ARB, β-blockers, and CCB were protective factors.

### PSM Cohort

We used PSM to identify 1700 patients with comparable baseline characteristics in both groups (Supplementary Table 2). In the PSM cohort, RFA was associated with a reduced risk of major bleeding [OR 0.290 (95% CI, 0.143-0.589), *P<*0.001], thrombosis [OR 0.509 (95% CI, 0.287-0.902), *P=*0.019] and composite endpoint [OR 0.818 (95% CI, 0.675-0.991), *P=* 0.040] (Table 3). There were no significant differences between the two groups regarding total bleeding and all-cause death.

**Table 3:** Clinical Outcomes of RFA vs. no RFA in NVAF patients taking DOACs after propensity score matching

	No RFA(*n=*1700)	RFA(*n=*1700)	OR(95%CI)	*P* value
Total bleeding, n(%)	185(10.9)	165(9.7)	0.880(0.705-1.099)	0.259
Major Bleeding, n(%)	34(2.0)	10(0.6)	0.290(0.143-0.589)	<0.001
Thrombosis, n(%)	35(2.1)	18(1.1)	0.509(0.287-0.902)	0.019
All-cause death, n(%)	52(3.1)	47(2.8)	0.901(0.604-1.345)	0.610
Composite outcome^a^	266(15.6)	224(13.2)	0.818(0.675-0.991)	0.040

*NVAF* Non-valvular atrial fibrillation, *RFA* Radiofrequency ablation, *OR* Odds ratio, *95%CI* 95% Confidence interval

^a^Composite outcome included all-cause death, thrombosis and total bleeding

### Subgroup analysis

We performed a subgroup analysis of the clinical outcomes of patients taking different DOACs (dabigatran, rivaroxaban) in the RFA group and no RFA group, respectively.

#### RFA group

Supplementary Table 3 is baseline information on the administration of rivaroxaban and dabigatran in patients with NVAF in the RFA group. We identified 773 patients in each group with comparable baseline characteristics with PSM. In the PSM cohort, dabigatran was associated with reduced all-cause death in patients with RFA of NVAF [OR 0.420 (95% CI, 0.212-0.831), *P=*0.010]. There were no significant differences between rivaroxaban and dabigatran for major bleeding, total bleeding, thrombosis, and composite endpoints (Supplementary Table 4).

#### No RFA group

Supplementary Table 5 is baseline information for NVAF patients taking rivaroxaban and dabigatran in the no RFA group. We identified 1301 patients in each group with comparable baseline characteristics with PSM. In the PSM cohort, rivaroxaban was associated with a reduction in major bleeding [OR 0.521 (95% CI, 0.403-0.673), *P<*0.001], total bleeding [OR 0.114 (95% CI, 0.049-0.266), *P<*0.001], and the composite endpoint [OR 0.659 (95% CI, 0.535-0.811), *P<*0.001]. There was no significant difference between rivaroxaban and dabigatran regarding thrombosis and all-cause death (Supplementary Table 6).

## Discussion

Our study is a multicenter retrospective cohort study based on 15 hospitals in China to investigate the difference in predictive outcomes between patients undergoing RFA and patients without RFA taking DOACs. Our study had the following findings: (1) During a mean follow-up of 10 months, RFA significantly affected major bleeding, thrombosis, and composite endpoints in NVAF patients taking DOACs. (2) Patients who underwent RFA had a reduced risk of major bleeding, thrombosis, and composite endpoints compared to patients without RFA. (3) Patients taking rivaroxaban versus dabigatran for RFA did not affect prognostic outcomes. (4) The risk of total bleeding, major bleeding, and composite endpoints was lower in patients without RFA taking rivaroxaban than on dabigatran.

Inconsistent results have been found in different studies regarding the benefits of ablation in patients with AF. In a Meta-analysis of 11 randomized controlled trials, CA of AF was found to reduce AF recurrence and improve quality of life compared with antiarrhythmic drug therapy but failed to change the risk of all-cause death, stroke, or TIA [28]. In contrast, a Meta-analysis of 9 studies by Saglietto et al. [29] found that CA reduced the risk of death, stroke, and hospitalization for heart failure compared to drug therapy alone. However, the benefits of ablation for patients with AF have been reported in several national observational studies in recent years. Mansour et al. [30] analyzed claims data and found a lower risk of thromboembolic events and cardiovascular hospitalization in patients with AF treated with CA compared with patients taking antiarrhythmic drugs. Srivatsa et al. [31] found that ablation was associated with lower mortality, ischemic stroke, and hemorrhagic stroke in a US population-based study of hospitalized patients with NVAF. A propensity-matched study by Saliba et al. [32] using a computerized database of the largest health maintenance organization in Israel, found that CA of AF was associated with reduced stroke/TIA and mortality in patients with higher CHA2DS2-Vasc scores. A study by Kim et al. [33] using the Korean National Health Insurance (NHIS) database showed that CA significantly reduced the risk of ischemic stroke and major bleeding compared with drug therapy.

However, the benefit of RFA for NVAF patients taking DOACs is currently poorly reported. Ding et al. [34] analyzed phase II/III data from the GLORIA-AF (GLORIA-AF is a prospective, observational, global registry programme of patients from 935 centers across 38 participating countries in Asia, Europe, North America, Latin America, and Africa/Middle East.) found that early AF ablation was associated with all-cause death and the composite endpoints of stroke, major bleeding, and all-cause death in patients treated with NOAC compared with drug therapy alone. In contrast to the previous study, we investigated the difference in outcomes of DOACs in NVAF patients who underwent RFA and those without RFA in 15 centers in China. We found that RFA was significantly associated with a reduction in the composite endpoint of bleeding, thrombosis. Still, we found that RFA was significantly associated with a reduction in major bleeding and thrombosis. The discrepancy may be due to the small number of patients with NVAF who underwent ablation, 445, including Ding et al. [34], and nearly 30% of these patients did not receive NOAC treatment. It may also be because Ding et al. included patients with generally higher age, BMI, and CHA2DS2-VASc scores, which may have had a greater impact on the results. In addition, the covariates included in the analysis in this study differ from those in Ding's study [34], which may impact the results after adjusting for confounding factors. RFA reduces the incidence of thrombosis possibly by restoring and maintaining sinus rhythm (SR) in patients with NVAF, thereby restoring atrial contraction, which may facilitate a reduction in the prothrombotic state, leading to a reduced risk of thrombosis [35]^.^ In addition, the reduced thrombotic risk in NVAF patients receiving RFA may be accompanied by a reduction in DOAC use, which consequently reduces the risk of major bleeding and the occurrence of composite endpoints.

We analyzed the factors influencing total bleeding, major bleeding, thrombosis, all-cause death, as well as composite endpoints and found that BMI was a protective factor for major bleeding, total bleeding, all-cause death, and composite endpoints, which is consistent with the results of our previous study [36]. Higher BMI was associated with lower major bleeding, total bleeding, and better survival. Our study identified combined heart failure and vascular disease risk factors for a composite endpoint in patients with NVAF. In contrast, Ding's study found a poorer prognosis in patients with greater age, decreased renal function, diabetes, coronary artery disease, heart failure, prior thromboembolism, prior bleeding, peripheral arterial disease, chronic obstructive pulmonary disease, and treatment with antiplatelet agents, digoxin, and diuretics. The inconsistent risk factors may be due to (1) the different populations included. Ding et al. [34] All patients newly diagnosed with AF (<3 months before the baseline visit) were from multiple countries. However, the present study is a multicenter retrospective study in China and does not define the time to diagnose NVAF. (2) Different confounding factors that may impact the analysis results were incorporated.

We performed separate subgroup analyses for patients in the RFA and no RFA groups. All-cause death among patients who underwent RFA was significantly lower in patients with dabigatran compared with rivaroxaban. In contrast, major bleeding, total bleeding, and composite endpoints were significantly lower in patients with rivaroxaban compared to dabigatran in the no RFA group. This suggests that in patients with NVAF undergoing RFA, postoperative use of dabigatran or rivaroxaban has similar efficacy, but there is a better survival benefit with dabigatran. However, patients with NVAF without RFA appear to benefit more from rivaroxaban. There is growing evidence that RFA is beneficial for the prognosis of patients with NVAF and that ablation should be considered early in AF, as success rates decrease with treatment delay [37].

Our study is the first multicenter retrospective cohort analysis in China with a large sample size exploring the differences between major bleeding, total bleeding, thrombosis, all-cause death, and the composite endpoints of bleeding, thrombosis, and all-cause death in patients with and without RFA of NVAF using DOACs, with a large patient base covering the full age range. Also, our study has several limitations: (1) Due to the retrospective nature of this study, information on the results may be incomplete. (2) Due to the majority of elderly patients, it is difficult to avoid unclear or confusing memory during follow-up. (3) Adherence to prescribed DOACs was not assessed.

## Conclusion

In patients with NVAF treated with DOACs, RFA was negatively associated with the risk of major bleeding, thrombosis, and the composite endpoint of bleeding, thrombosis, and all-cause death but was not associated with total bleeding or all-cause death. Patients with RFA of NVAF have a better prognosis with DOACs than those with NVAF without RFA.

## Supplementary Information

**Additional file1:Table 1.** List of 15 multi-center hospitals, **Table 2.** Baseline characteristics after propensity score matching, **Table 3.** Baseline characteristics of patients with NVAF in the RFA group taking rivaroxaban and dabigatran before and after matching, **Table 4.** Clinical Outcomes of DOACs in NVAF patients with RFA after propensity score matching, **Table 5.** Baseline characteristics of patients with NVAF in the no RFA group taking rivaroxaban and dabigatran before and after matching, **Table 6.** Clinical Outcomes of DOACs in NVAF patients with no RFA after propensity score matching, **Fig. 1.** Sub-center Distribution Map, **Fig. 2.** Association of the RFA and Potential Risk Factors with Total Bleeding in NVAF Patients, **Fig. 3.** Association of the RFA and Potential Risk Factors with Major Bleeding in NVAF Patients, **Fig. 4.** Association of the RFA and Potential Risk Factors with Thrombosis in NVAF Patients, **Fig. 5.** Association of the RFA and Potential Risk Factors with All-caused Deaths in NVAF Patients, **Fig. 6.** Association of the RFA and Potential Risk Factors with Composite outcome in NVAF Patients.

## Data Availability

All data relevant to the study are included in the article as supplementary information.
