# Supplementary material for: Safety and efficacy of direct oral anticoagulation in patients with and without radiofrequency ablation of non-valvular atrial fibrillation: a multicenter retrospective cohort study
Source: Thromb J. 2023 Apr 5;21:37. doi: 10.1186/s12959-023-00483-6 (PMC10074713; doi:10.1186/s12959-023-00483-6)
Supplement: Supplementary file 1 — Additional file1:Table 1. List of 15 multi-center hospitals, Table 2. Baseline characteristics after propensity score matching, Table 3. Baseline characteristics of patients with NVAF in the RFA group taking rivaroxaban and dabigatran before and after matching, Table 4. Clinical Outcomes of DOACs in NVAF patients with RFA after propensity score matching, Table 5. Baseline characteristics of patients with NVAF in the no RFA group taking rivaroxaban and dabigatran before and after matching, Table 6. Clinical Outcomes of DOACs in NVAF patients with no RFA after propensity score matching, Fig. 1. Sub-center Distribution Map, Fig. 2. Association of the RFA and Potential Risk Factors with Total Bleeding in NVAF Patients, Fig. 3. Association of the RFA and Potential Risk Factors with Major Bleeding in NVAF Patients, Fig. 4. Association of the RFA and Potential Risk Factors with Thrombosis in NVAF Patients, Fig. 5. Association of the RFA and Potential Risk Factors with All-caused Deaths in NVAF Patients, Fig. 6. Association of the RFA and Potential Risk Factors with Composite outcome in NVAF Patients. [file 12959_2023_483_MOESM1_ESM.docx]

**Table 1** List of 15 multi-center hospitals

| Number | Institutes | Investigators |
| --- | --- | --- |
| 1 | Shanxi Bethune Hospital | Ruijuan Li |
| 2 | Suining Central Hospital, Suining | Ping Gu |
| 3 | First Affiliated Hospital of Xi'an Jiaotong University | Qiaowei Zheng |
| 4 | People’s Hospital of He’nan University of Chinese Medicine | Xiumei Liu |
| 5 | Affiliated Fuzhou First Hospital of Fujian Medical University | Hengfen Dai |
| 6 | Pingtan County General Laboratory Area Hospital | Xiangsheng Lin |
| 7 | Huaihe Hospital of Henan University, kaifeng | Yuxin Liu |
| 8 | Shengjing hospital of China Medical University | Xiaoming Du |
| 9 | Department of Pharmacy, the First Affiliated Hospital of Bengbu Medical College | Jun Su |
| 10 | The first people‘s Hospital of Changde City | Wang Zhang |
| 11 | Affiliated Qingdao Third People's Hospital | Min Zhang |
| 12 | The Second Affiliated Hospital of Soochow University | Zhu Zhu |
| 13 | Zhangzhou affiliated Hospital of Fujian Medical University | Xiaohong Huang |
| 14 | Wuhan Asian Heart Hospital | Nianxu Huang |
| 15 | Fujian Medical University Union Hospital | Jinhua Zhang |
| 16 |  |  |

**Table 2** Baseline characteristics after propensity score matching

|  | No radiofrequency ablation (n=1700) | Radiofrequency ablation (n=1700) | SMD |
| --- | --- | --- | --- |
| Age, years, mean (SD) | 62.5(10.7) | 62.5(9.5) | 0.005 |
| Sex, male, n (%) | 1004(59.1) | 1001(58.9) | -0.004 |
| BMI, mean (SD) | 24.8(3.8) | 24.7(3.4) | -0.032 |
| Smoking, n(%) | 1052(61.9) | 1044(61.4) | -0.011 |
| Alcohol, n(%) | 641(37.7) | 634(37.3) | -0.008 |
| Comorbidities, n(%) |  |  |  |
| Hypertension | 909(53.5) | 905(53.2) | -0.005 |
| Diabetes mellitus | 294(17.3) | 284(16.7) | -0.017 |
| Congestive heart failure | 17(1.0) | 10(0.6) | -0.067 |
| Coronary heart disease | 28(1.6) | 20(1.2) | -0.045 |
| Renal insufficiency | 30(1.8) | 25(1.5) | -0.030 |
| Hypohepatia | 14(0.8) | 14(0.8) | 0.000 |
| Vascular disease | 68(4.0) | 59(3.5) | -0.036 |
| Laboratories |  |  |  |
| TBIL, umol/L, mean (SD) | 15.9(6.3) | 16.1(6.3) | 0.032 |
| ALT, IU/L, mean (SD) | 27.0(27.3) | 26.3(24.8) | -0.032 |
| AST, IU/L, mean (SD) | 39.4(25.7) | 38.6(34.0) | -0.027 |
| Creatinine, umol/L, mean (SD) | 79.6(20.9) | 78.9(18.2) | -0.038 |
| PLT, 109/L, mean (SD) | 182.4(52.4) | 181.5(46.9) | -0.018 |
| Hb, g/L, mean (SD) | 135.5(16.1) | 135.3(13.3) | -0.018 |
| Combined medication, n(%) |  |  |  |
| Antiplatelet drugs | 476(28.0) | 479(28.2) | 0.004 |
| PPI | 1173(69.0) | 1185(69.7) | -0.016 |
| Statins | 853(50.2) | 862(50.7) | -0.011 |
| Amiodarone | 297(17.5) | 297(17.5) | 0.000 |
| H2-blockers | 581(34.2) | 606(35.6) | 0.032 |
| ACEI | 428(25.2) | 436(25.6) | 0.011 |
| ARB | 447(26.3) | 462(27.2) | -0.021 |
| β-Blockers | 1162(68.4) | 1195(70.3) | -0.041 |
| CCB | 599(35.2) | 554(32.6) | -0.006 |
| Diltiazem | 278(16.4) | 199(11.7) | 0.035 |
| Digoxin | 183(10.8) | 185(10.9) | 0.004 |
| CHA2DS2-VASc, Mean(SD) | 1.7(1.2) | 1.7(1.2) | -0.011 |
| HAS-BLED, Mean(SD) | 1.2(0.9) | 1.2(0.9) | -0.013 |

SMD: Standardized Mean; Difference; SD: standard deviation; BMI: Body Mass Index; TBIL: Total Bilirubin; ALT: Alanine Transaminase; AST: Aspartate transaminase; PLT: Platelet count; Hb: Hemoglobin; PPI: Proton pump inhibitor; ACEI: Angiotensin converting enzyme inhibitor; ARB: Angiotensin receptor blockers; CCB: Calcium Calcium Entry Blockers

**Table 3** Baseline characteristics of patients with NVAF in the RFA group taking rivaroxaban and dabigatran before and after matching

|  | Unmatched Cohort | | | Matched Cohort | | |
| --- | --- | --- | --- | --- | --- | --- |
|  | Dabigatran (n=1658) | Rivaroxaban (n=1003) | SMD | Dabigatran (n=773) | Rivaroxaban (n=773) | SMD |
| Age, years, mean (SD) | 58.9(10.5) | 60.2(10.4) | -0.127 | 60.0(10.5) | 59.7(10.5) | 0.031 |
| Sex, male, n (%) | 1069(64.5) | 665(66.3) | -0.038 | 500(64.7) | 514(66.5) | -0.038 |
| BMI, mean (SD) | 24.8(3.4) | 24.8(3.3) | -0.045 | 24.6(3.4) | 24.8(3.3) | 0.003 |
| Smoking, n(%) | 1200(72.4) | 530(52.8) | 0.379 | 428(55.4) | 447(57.8) | -0.060 |
| Alcohol, n(%) | 731(44.1) | 373(37.2) | 0.059 | 276(35.7) | 290(37.5) | -0.059 |
| Comorbidities, n(%) |  |  |  |  |  |  |
| Hypertension | 775(46.7) | 487(46.8) | -0.036 | 358(46.3) | 368(47.6) | -0.026 |
| Diabetes mellitus | 199(12.0) | 162(16.2) | -0.128 | 110(14.2) | 107(13.8) | 0.012 |
| Congestive heart failure | 1(0.1) | 9(0.9) | -0.341 | 1(0.1) | 2(0.3) | -0.053 |
| Coronary heart disease | 5(0.3) | 24(2.4) | -0.381 | 5(0.6) | 4(0.5) | 0.024 |
| Renal insufficiency | 17(1.0) | 8(0.8) | 0.023 | 8(1.0) | 8(1.0) | 0.000 |
| Hypohepatia | 9(0.5) | 5(0.5) | 0.006 | 6(0.8) | 5(0.6) | 0.018 |
| Vascular disease | 22(1.3) | 38(3.8) | -0.215 | 17(2.2) | 17(2.2) | 0.000 |
| Laboratories |  |  |  |  |  |  |
| TBIL, umol/L, mean (SD) | 15.6(5.1) | 15.1(7.0) | 0.090 | 15.5(5.2) | 15.5(6.8) | -0.001 |
| ALT, IU/L, mean (SD) | 24.9(20.5) | 27.3(24.0) | -0.118 | 25.5(17.7) | 26.5(21.8) | -0.046 |
| AST, IU/L, mean (SD) | 36.0(25.6) | 34.4(34.0) | 0.064 | 36.6(30.5) | 36.3(36.6) | 0.010 |
| Creatinine, umol/L, mean (SD) | 77.9(14.9) | 79.0(20.3) | -0.074 | 77.9(16.0) | 78.5(19.3) | -0.044 |
| PLT, 109/L, mean (SD) | 181.4(44.2) | 197.5(56.7) | -0.363 | 188.7(47.4) | 188.2(52.0) | 0.010 |
| Hb, g/L, mean (SD) | 137.6(13.1) | 139.4(16.3) | -0.131 | 138.2(13.5) | 138.3(15.0) | -0.009 |
| Combined medication, n(%) |  |  |  |  |  |  |
| Antiplatelet drugs | 534(32.2) | 216(21.5) | 0.139 | 191(24.7) | 186(24.1) | 0.014 |
| PPI | 460(27.7) | 764(76.2) | -0.076 | 589(76.2) | 569(73.6) | 0.058 |
| Statins | 814(49.1) | 373(37.2) | 0.313 | 338(43.7) | 330(42.7) | 0.016 |
| Amiodarone | 280(16.9) | 247(24.6) | 0.238 | 143(18.5) | 150(19.4) | 0.021 |
| H2-blockers | 620(37.4) | 223(22.2) | -0.206 | 227(29.4) | 221(28.6) | -0.024 |
| ACEI | 413(24.9) | 162(16.2) | 0.202 | 161(20.8) | 152(19.7) | 0.027 |
| ARB | 401(24.2) | 242(24.1) | 0.001 | 182(23.5) | 192(24.8) | -0.030 |
| β-Blockers | 1177(71.0) | 562(56.0) | 0.329 | 485(62.7) | 492(63.6) | -0.020 |
| CCB | 550(33.2) | 279(27.8) | 0.114 | 235(30.4) | 237(30.7) | -0.005 |
| Diltiazem | 261(15.7) | 131(13.1) | 0.074 | 120(15.5) | 124(16.0) | -0.014 |
| Digoxin | 156(9.4) | 84(8.4) | 0.035 | 65(8.4) | 67(8.7) | -0.009 |
| CHA2DS2-VASc, Mean(SD) | 1.3(1.1) | 1.5(1.2) | -0.143 | 1.4(1.2) | 1.4(1.2) | 0.029 |
| HAS-BLED, Mean(SD) | 1.0(0.9) | 1.0(0.9) | -0.062 | 1.0(0.9) | 1.0(0.9) | 0.023 |

NVAF: Non-Valvular Atrial Fibrillation; RFA: Radiofrequency Ablation; SMD: Standardized Mean; Difference; SD: standard deviation; BMI: Body Mass Index; TBIL: Total Bilirubin; ALT: Alanine Transaminase; AST: Aspartate transaminase; PLT: Platelet count; Hb: Hemoglobin; PPI: Proton pump inhibitor; ACEI: Angiotensin converting enzyme inhibitor; ARB: Angiotensin receptor blockers; CCB: Calcium Calcium Entry Blockers

**Table 4** Clinical Outcomes of DOACs in NVAF patients with RFA after propensity score matching

|  | Dabigatran (n=773) | Rivaroxaban (n=773) | P value | OR(95%CI)） |
| --- | --- | --- | --- | --- |
| Total Bleeding, n(%) | 82(10.6) | 85(11.0) | 0.806 | 0.961(0.679-1.324) |
| Major Bleeding, n(%) | 3(0.4) | 5(0.6) | 0.478 | 0.598(0.143-2.513) |
| Thrombosis, n(%) | 11(1.4) | 7(0.9) | 0.343 | 1.580(0.609-4.097) |
| All-cause Death, n(%) | 12(1.6) | 28(3.6) | 0.010 | 0.420(0.212-0.831) |
| Composite outcome^a^, n(%) | 114(14.7) | 101(13.1) | 0.339 | 0.869(0.651-1.159) |

NVAF: Non-Valvular Atrial Fibrillation; RFA: Radiofrequency Ablation; OR: Odds Ratio; 95%CI: 95% Confidence Interval

a: Composite outcome included all-cause death, thrombosis and total bleeding.

**Table 5** Baseline characteristics of patients with NVAF in the no RFA group taking rivaroxaban and dabigatran before and after matching

|  | Unmatched Cohort | | | Matched Cohort | | |
| --- | --- | --- | --- | --- | --- | --- |
|  | Dabigatran (n=1834) | Rivaroxaban (n=1642) | SMD | Dabigatran (n=1301) | Rivaroxaban (n=1301) | SMD |
| Age, years, mean (SD) | 67.2(12.1) | 67.0(10.4) | -0.025 | 66.6(11.7) | 514(66.5) | 0.008 |
| Sex, male, n (%) | 1020(55.6) | 865(52.7) | -0.059 | 714(54.9) | 701(53.9) | -0.020 |
| BMI, mean (SD) | 24.1(3.8) | 24.7(3.8) | 0.161 | 24.4(3.9) | 24.8(3.3) | -0.010 |
| Smoking, n(%) | 975(53.2) | 1024(62.4) | 0.240 | 756(58.1) | 750(57.6) | -0.011 |
| Alcohol, n(%) | 628(34.2) | 632(38.5) | 0.110 | 468(36.0) | 446(34.3) | -0.034 |
| Comorbidities, n(%) |  |  |  |  |  |  |
| Hypertension | 1067(58.2) | 972(59.2) | 0.021 | 750(57.6) | 769(59.1) | 0.030 |
| Diabetes mellitus | 401(21.9) | 319(19.4) | -0.062 | 261(20.1) | 258(19.8) | -0.006 |
| Congestive heart failure | 170(9.3) | 29(1.8) | -0.569 | 28(2.2) | 28(2.2) | 0.000 |
| Coronary heart disease | 152(8.3) | 155(9.4) | 0.039 | 83(6.4) | 94(7.2) | 0.029 |
| Renal insufficiency | 172(9.4) | 99(0.6) | -0.141 | 101(7.8) | 93(7.1) | -0.026 |
| Hypohepatia | 57(3.1) | 30(1.8) | -0.096 | 32(2.5) | 29(2.2) | -0.017 |
| Vascular disease | 328(17.9) | 265(16.1) | -0.047 | 194(14.9) | 187(14.4) | -0.015 |
| Laboratories |  |  |  |  |  |  |
| TBIL, umol/L, mean (SD) | 16.7(10.0) | 17.6(8.0) | 0.117 | 17.5(10.8) | 15.5(6.8) | -0.046 |
| ALT, IU/L, mean (SD) | 28.1(36.8) | 28.7(46.7) | -0.012 | 28.5(40.5) | 26.5(21.8) | -0.009 |
| AST, IU/L, mean (SD) | 45.6(58.3) | 45.4(74.0) | -0.002 | 48.3(65.5) | 36.3(36.6) | -0.028 |
| Creatinine, umol/L, mean (SD) | 88.6(35.9) | 81.4(21.4) | -0.317 | 83.0(25.0) | 78.5(19.3) | -0.004 |
| PLT, 10^9^/L, mean (SD) | 183.3(63.1) | 183.2(68.8) | -0.001 | 181.5(62.1) | 188.2(52.0) | 0.014 |
| Hb, g/L, mean (SD) | 131.4(18.8) | 132.7(15.2) | 0.079 | 132.9(18.1) | 138.3(15.0) | -0.027 |
| Combined medication, n(%) |  |  |  |  |  |  |
| Antiplatelet drugs | 466(25.4) | 543(33.1) | 0.163 | 378(29.1) | 382(29.4) | 0.007 |
| PPI | 1086(59.2) | 1080(65.8) | 0.138 | 834(64.1) | 835(64.2) | 0.002 |
| Statins | 984(53.7) | 918(55.9) | 0.220 | 702(54.0) | 705(54.2) | 0.006 |
| Amiodarone | 286(15.6) | 272(16.6) | 0.045 | 218(16.8) | 220(16.9) | 0.005 |
| H2-blockers | 487(26.6) | 611(37.2) | 0.026 | 436(33.5) | 440(33.8) | 0.004 |
| ACEI | 424(23.1) | 453(27.6) | 0.100 | 328(25.2) | 318(24.4) | -0.017 |
| ARB | 509(27.8) | 441(26.9) | -0.020 | 374(28.7) | 354(27.2) | -0.035 |
| β-Blockers | 1191(64.9) | 1197(72.9) | 0.179 | 924(71.0) | 907(69.7) | -0.029 |
| CCB | 553(30.2) | 557(33.9) | 0.080 | 430(33.1) | 414(31.8) | -0.026 |
| Diltiazem | 272(14.8) | 284(17.3) | 0.065 | 219(16.8) | 219(16.8) | 0.000 |
| Digoxin | 229(12.5) | 190(11.6) | -0.029 | 162(12.5) | 152(11.7) | -0.024 |
| CHA2DS2-VASc, Mean(SD) | 2.6(1.6) | 2.4(1.5) | -1.006 | 2.4(1.4) | 1.4(1.2) | 0.018 |
| HAS-BLED, Mean(SD) | 1.5(1.0) | 1.5(0.9) | -0.047 | 1.5(0.9) | 1.0(0.9) | 0.003 |

NVAF: Non-Valvular Atrial Fibrillation; RFA: Radiofrequency Ablation; SMD: Standardized Mean; Difference; SD: standard deviation; BMI: Body Mass Index; TBIL: Total Bilirubin; ALT: Alanine Transaminase; AST: Aspartate transaminase; PLT: Platelet count; Hb: Hemoglobin; PPI: Proton pump inhibitor; ACEI: Angiotensin converting enzyme inhibitor; ARB: Angiotensin receptor blockers; CCB: Calcium Calcium Entry Blockers

**Table 6** Clinical Outcomes of DOACs in NVAF patients with no RFA after propensity score matching

|  | Dabigatran (n=1301) | Rivaroxaban (n=1301) | P value | OR(95%CI) |
| --- | --- | --- | --- | --- |
| Total Bleeding, n(%) | 181(13.9) | 101(7.8) | <0.001 | 0.521(0.403-0.673) |
| Major Bleeding, n(%) | 51(3.9) | 6(0.5) | <0.001 | 0.114(0.049-0.266) |
| Thrombosis, n(%) | 20(1.5) | 25(2.0) | 0.452 | 1.255(0.693-2.271) |
| All-cause Death, n(%) | 57(4.4) | 63(4.8) | 0.575 | 1.111(0.770-1.603) |
| Composite outcome^a^, n(%) | 255(19.6) | 180((13.8) | <0.001 | 0.659(0.535-0.811) |

NVAF: Non-Valvular Atrial Fibrillation; RFA: Radiofrequency Ablation; OR: Odds Ratio; 95%CI: 95% Confidence Interval

a: Composite outcome included all-cause death, thrombosis and total bleeding.


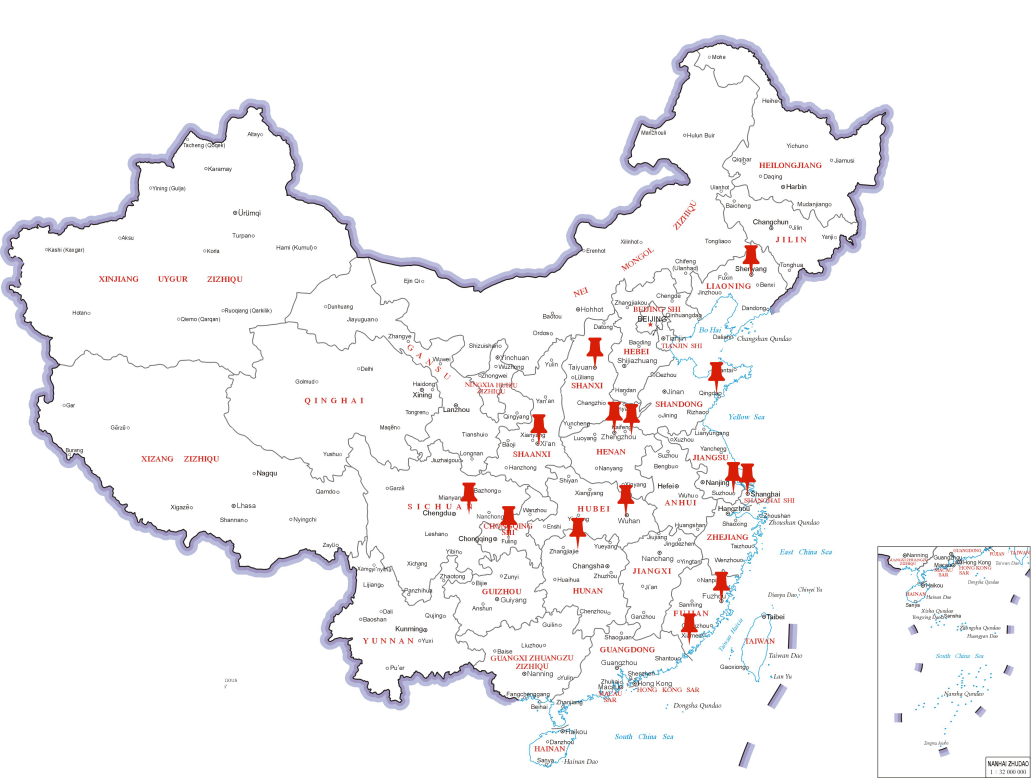


**Fig. 1** Sub-center Distribution Map

**
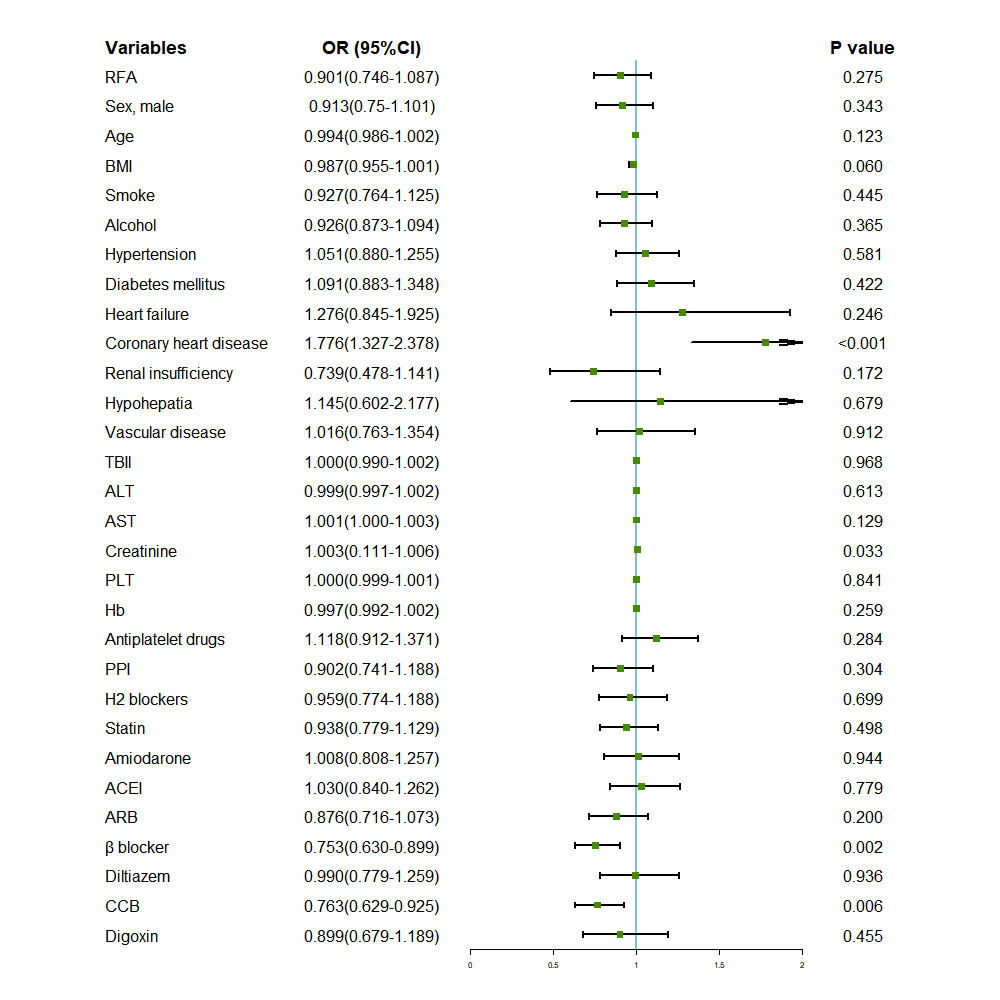
**

**Fig. 2** Association of the RFA and Potential Risk Factors with Total Bleeding in NVAF Patients

Notes: OR: Odds Ratio; 95%CI: 95% Confidence Interval; NVAF: non-valvular atrial fibrillation; RFA: Radiofrequency Ablation; BMI: Body Mass Index; TBIL: Total Bilirubin; ALT: Alanine Transaminase; AST: Aspartate transaminase; PLT: Platelet count; Hb: Hemoglobin; PPI: Proton pump inhibitor; ACEI: Angiotensin converting enzyme inhibitor; ARB: Angiotensin receptor blockers; CCB: Calcium Calcium Entry Blockers


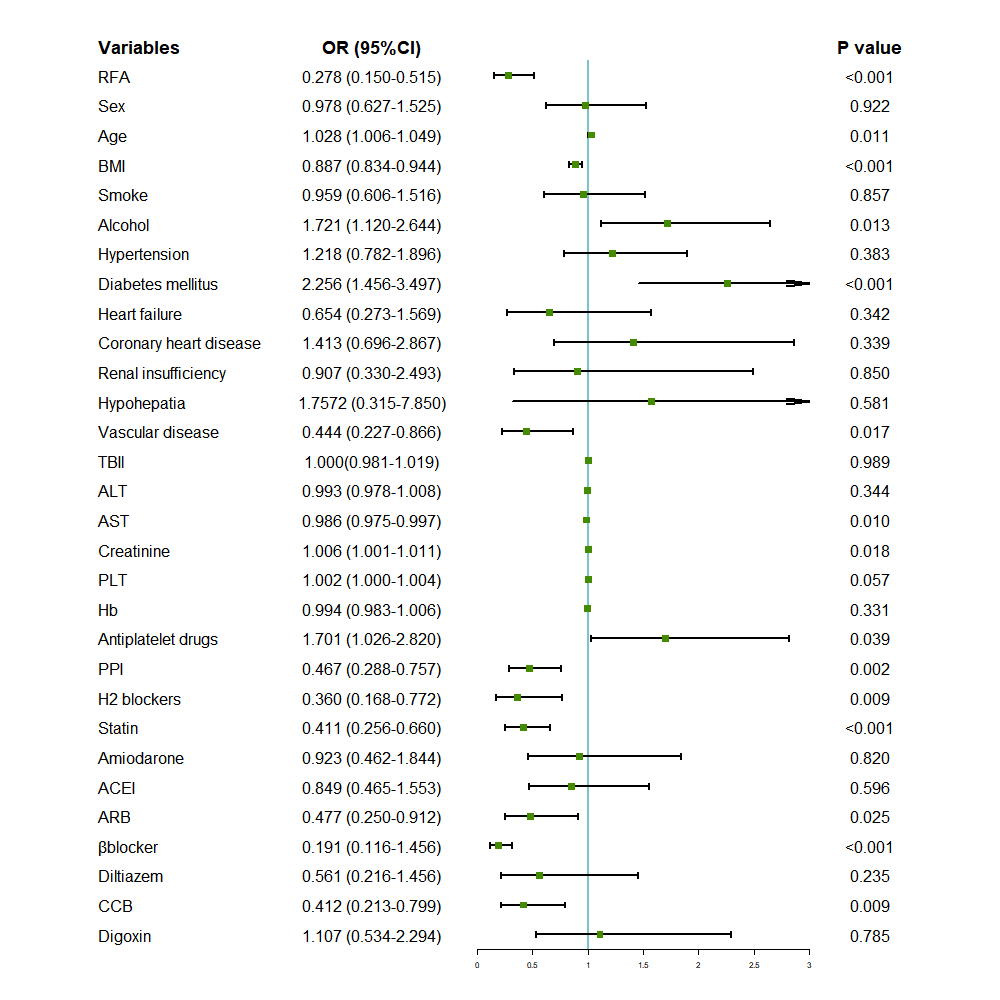


**Fig. 3** Association of the RFA and Potential Risk Factors with Major Bleeding in NVAF Patients

Notes: OR: Odds Ratio; 95%CI: 95% Confidence Interval; NVAF: non-valvular atrial fibrillation; RFA: Radiofrequency Ablation; BMI: Body Mass Index; TBIL: Total Bilirubin; ALT: Alanine Transaminase; AST: Aspartate transaminase; PLT: Platelet count; Hb: Hemoglobin; PPI: Proton pump inhibitor; ACEI: Angiotensin converting enzyme inhibitor; ARB: Angiotensin receptor blockers; CCB: Calcium Calcium Entry Blockers


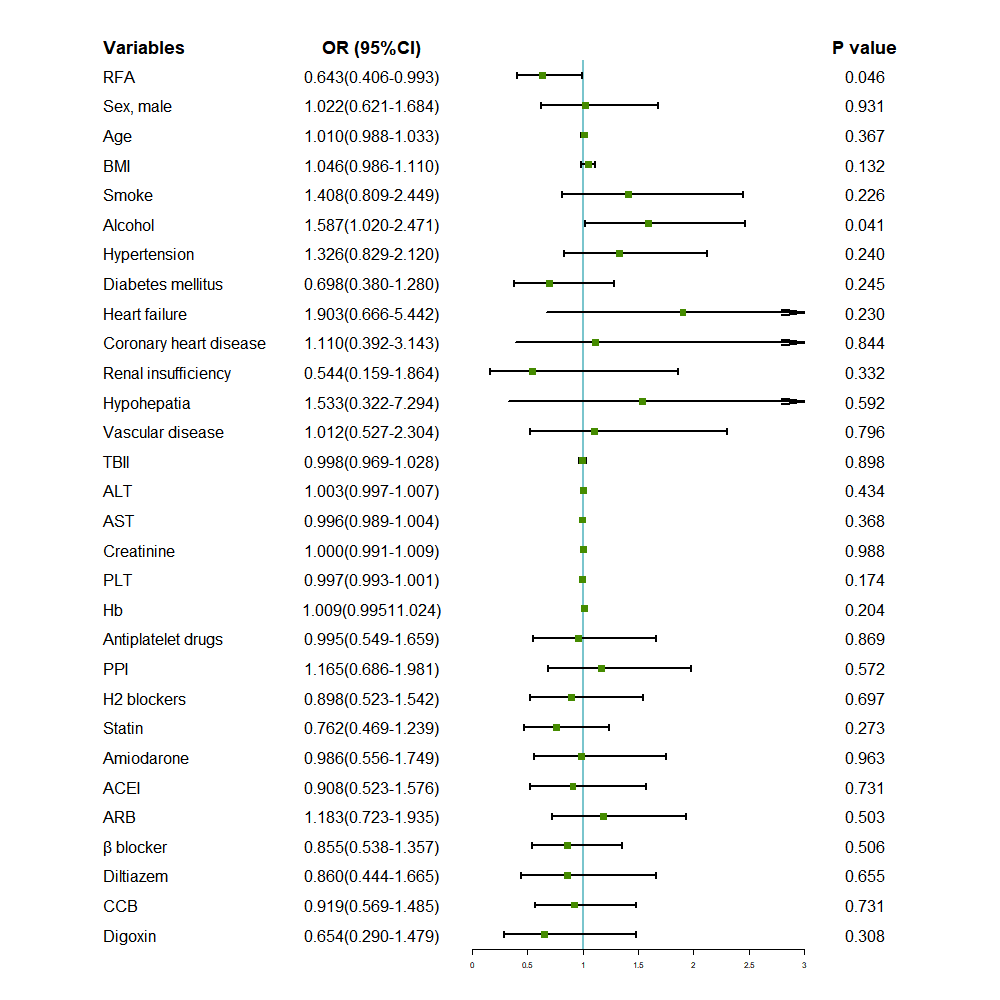


**Fig. 4** Association of the RFA and Potential Risk Factors with Thrombosis in NVAF Patients

Notes: OR: Odds Ratio; 95%CI: 95% Confidence Interval; NVAF: non-valvular atrial fibrillation; RFA: Radiofrequency Ablation; BMI: Body Mass Index; TBIL: Total Bilirubin; ALT: Alanine Transaminase; AST: Aspartate transaminase; PLT: Platelet count; Hb: Hemoglobin; PPI: Proton pump inhibitor; ACEI: Angiotensin converting enzyme inhibitor; ARB: Angiotensin receptor blockers; CCB: Calcium Calcium Entry Blockers

**
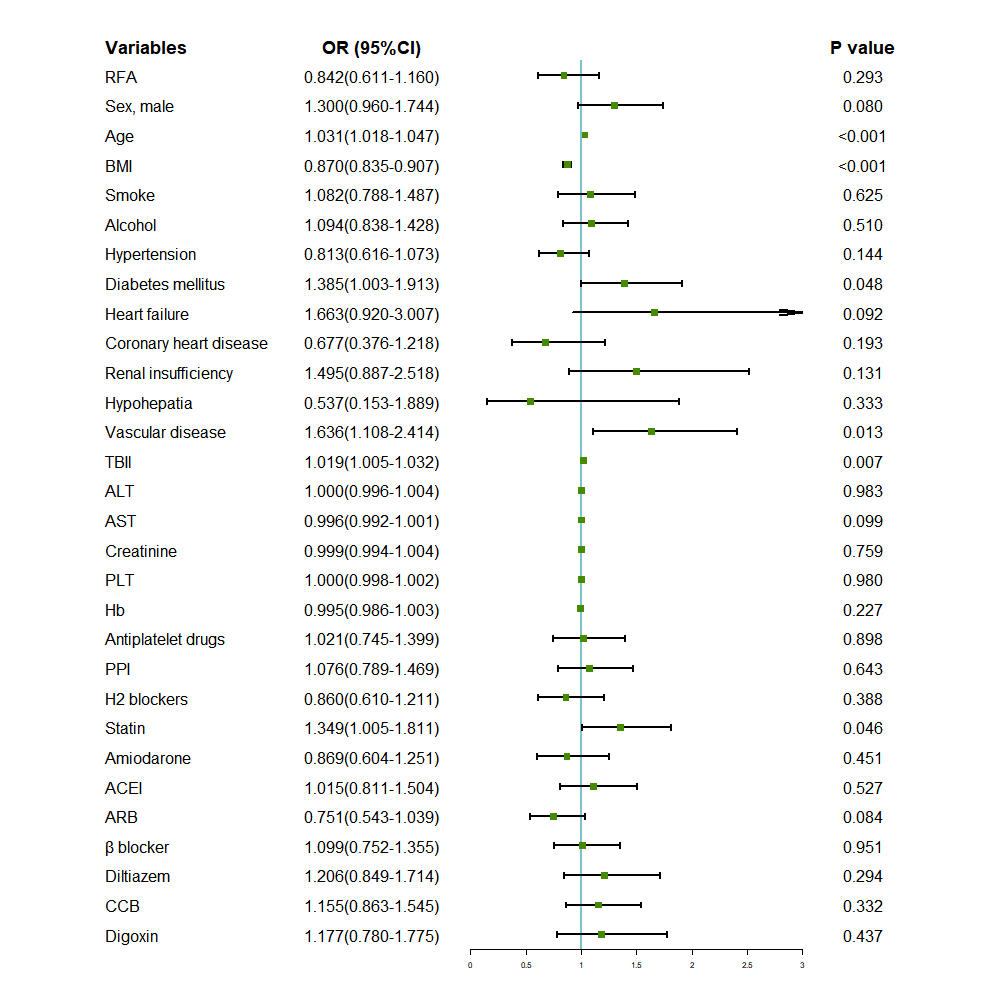
**

**Fig. 5** Association of the RFA and Potential Risk Factors with All-caused Deaths in NVAF Patients

Notes: OR: Odds Ratio; 95%CI: 95% Confidence Interval; NVAF: non-valvular atrial fibrillation; RFA: Radiofrequency Ablation; BMI: Body Mass Index; TBIL: Total Bilirubin; ALT: Alanine Transaminase; AST: Aspartate transaminase; PLT: Platelet count; Hb: Hemoglobin; PPI: Proton pump inhibitor; ACEI: Angiotensin converting enzyme inhibitor; ARB: Angiotensin receptor blockers; CCB: Calcium Calcium Entry Blockers


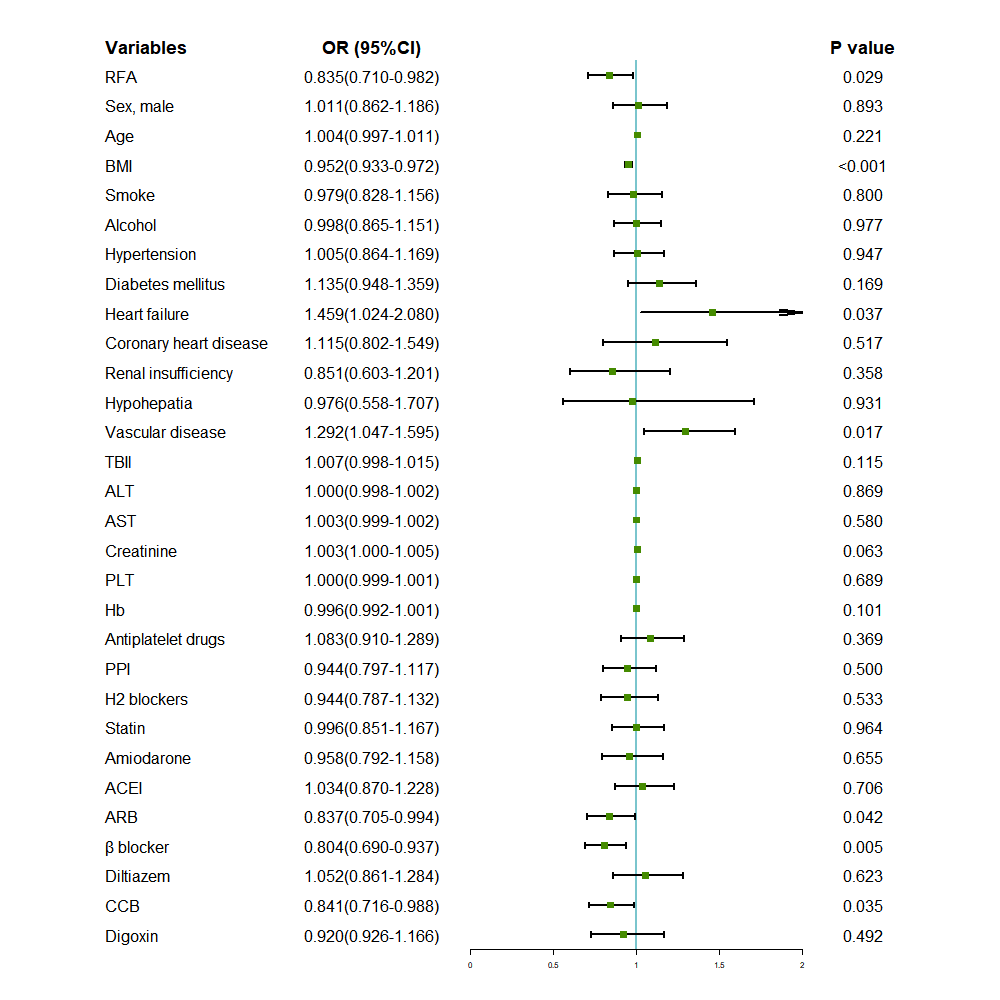


**Fig. 6** Association of the RFA and Potential Risk Factors with Composite outcome in NVAF Patients

Notes: OR: Odds Ratio; 95%CI: 95% Confidence Interval; NVAF: non-valvular atrial fibrillation; RFA: Radiofrequency Ablation; BMI: Body Mass Index; TBIL: Total Bilirubin; ALT: Alanine Transaminase; AST: Aspartate transaminase; PLT: Platelet count; Hb: Hemoglobin; PPI: Proton pump inhibitor; ACEI: Angiotensin converting enzyme inhibitor; ARB: Angiotensin receptor blockers; CCB: Calcium Calcium Entry Blockers
